# Ketogenic diet for anxiety disorders: Dietary regimen for relapse prevention?

**DOI:** 10.1192/j.eurpsy.2021.509

**Published:** 2021-08-13

**Authors:** A. Włodarczyk, W. Cubała

**Affiliations:** Psychiatry, Medical University of Gdansk, gdansk, Poland

**Keywords:** Anxiety, Ketogenic Diet, GABA

## Abstract

**Introduction:**

Anxiety disorders are a mental disorder that is widespread all over the world and are associated with a high burden of the disease. While poor dietary habits are common in depression, they can lead to worsening of the illness or prolong the treatment, which also leads to a higher risk of developing chronic diseases.

**Objectives:**

With the array of the treatment modalities including monoaminergic antidepressants, benzodiazepines, psychotherapy, there is the unmet need for the effective treatment of anxiety disorders resulting in full remission and relapse prevention. Benzodiazepines can quickly resolve anxiety due to their positive allosteric GABA modulation mechanism of action. Although, they are not recommended for chronic use.

**Methods:**

Ketogenic diet(KD) may be hypothesized as the promising strategy impacting treatment strategies, in particularly facilitating full remission, recovery and preventing relapses. In this popular high-fat diet, where daily calories intake is consists in at least 70% from fat, up to 25 % from protein and as little sugar as possible is mainly known for its helpful role in drug resistant epilepsy treatment, glucose levels balance or fast way for weight-loss.

**Results:**

Could be effective in anxiety disorders treatment due to its possible role in GABA:glutamate balance change in favor of GABA levels, which may enhance the anxiolytic effect in sustaining remission and preventing relapse.

**Conclusions:**

KD in some anxiety disorders may provide a rewarding outcome, but more research is needed. The evidence mentioned in this paper should encourage psychiatrists to recommend KD as advice somewhere between psychotherapy, pharmacology or as an add-on to those two.

